# Flexible endoscopy is enough diagnostic prior to loop ileostomy reversal

**DOI:** 10.1007/s00384-020-03766-w

**Published:** 2020-10-13

**Authors:** S. Lindner, K. von Rudno, J. Gawlitza, J. Hardt, F. Sandra-Petrescu, S. Seyfried, P. Kienle, C. Reissfelder, A. Bogner, F. Herrle

**Affiliations:** 1 Department of Surgery, University Hospital Mannheim, University Medical Centre Mannheim (UMM), Medical Faculty Mannheim, University of Heidelberg, Theodor-Kutzer-Ufer 1-3, 68167 Mannheim, Germany; 2 Department of Visceral, Thoracic and Vascular Surgery, University Hospital Carl Gustav Carus, University of Dresden, Dresden, Germany; 3 Department of Radiology, University Hospital Mannheim, Medical Faculty Mannheim, University of Heidelberg, Mannheim, Germany; 4 Department of Surgery, Theresienkrankenhaus Mannheim, Mannheim, Germany

**Keywords:** Ileostomy reversal, Asymptomatic anastomotic leak, Diagnostic test accuracy, Flexible endoscopy, Contrast enema

## Abstract

**Purpose:**

This study investigates whether contrast enema (CE) and flexible endoscopy (FE) should be performed routinely after low anterior resection (LAR) before ileostomy reversal. Additionally, the impact of previous anastomotic leakage (AL) on diagnostic test accuracy (DTA) was assessed.

**Methods:**

This is a retrospective analysis of prospectively collected tertiary care data of two centers. Consecutive rectal cancer patients undergoing LAR with loop ileostomy formation were included. Before ileostomy reversal, all patients were assessed by CE and FE. DTA of FE and CE for asymptomatic AL in patients who had previously suffered from clinically relevant AL (group 1) compared with those without apparent AL after LAR (group 0) were assessed separately.

**Results:**

Two hundred ninety-three patients were included in the analysis, 86 in group 1 and 207 in group 0. Overall sensitivity for detection of asymptomatic AL was 76% (FE) and 60% (CE). Specificity was 100% for both tests. DTA of FE was equal or superior to CE in all subgroups. Prevalence of asymptomatic AL at the time of testing was 1.4% in group 0 and 25.6% in group 1.

**Conclusion:**

Flexible endoscopy is the more accurate diagnostic test for the detection of asymptomatic anastomotic leaks prior to ileostomy reversal. Contrast enema showed no gain of information. In the group without complications after the initial rectal resection, 104 must be tested to find one leak prior to reversal. In those patients, routine diagnostic testing additional to digital rectal examination may be questioned.

## Introduction

Fecal diversion through formation of a loop ileostomy after low anterior resection (LAR) in rectal cancer patients is a widely used and evidence-based routine to attenuate consequences of a postoperative anastomotic leak (AL) [1]. Before reversal of the ileostomy, however, clinicians must be sure there is no asymptomatic AL that may become clinically apparent after restoration of intestinal continuity. Although this represents a frequent clinical situation, still no clear consensus exists on how to best assess the integrity of the colorectal anastomosis. Common examination techniques are digital rectal examination (DRE), flexible or rigid endoscopy (FE), and contrast enema radiography (CE) [2]. However, the routine use of CE for this indication is debatable. While some authors see benefits in its routine performance [3–6], others request limiting its use to selected patients [7–16]. It should be noted that in all studies promoting its routine use, CE was not compared with an endoscopic examination such as flexible endoscopy (FE) or rigid proctoscopy. Studies that routinely performed endoscopic examinations were more critical towards an additional routine CE. These findings could arise from the general need for a diagnostic procedure and a superiority of endoscopy over CE. However, differences in patient cohorts might also have influenced findings. Suggested characteristics of individual patients who could potentially benefit from CE were complications after LAR [10, 12, 15], abnormal findings in DRE or proctoscopy [8, 11], and clinical suspicion for a leak [7, 11, 13, 16]. However, to our knowledge, no current study formally analyzed those criteria.

The primary aim of this study was to investigate if routine CE adds clinically relevant information to a routinely performed FE by analyzing concordance and diagnostic test accuracy. In addition, the influence of a history of clinically apparent AL after LAR on the diagnostic test accuracy of CE and FE was assessed.

## Materials and methods

### Patients

Inclusion criteria were previously performed elective low anterior resection for rectal cancer and simultaneous fecal diversion through a loop ileostomy. At the time of testing, patients had to be in the preparation process of ileostomy reversal, showing no clinical sings of an anastomotic leak.

### Index tests

Contrast enema (CE) and flexible endoscopy (FE) are the index tests of this study. CE was performed by a radiologist using either antegrade or retrograde contrast enema technique. For antegrade enema, iodine contrast agent diluted in 1:1 ratio with lukewarm water was instilled in the efferent limb of the ileostomy via an unblocked 12 Charrière Foley catheter. The contrast medium was instilled freely with an approximate pressure of 70 cmH_2_0 for image acquisition. For retrograde enema, the same contrast agent was instilled through the anus.

FE was performed with a flexible endoscope. Prior to FE, inspection of the perianal region and digital rectal examination were performed. Then a flexible endoscope was inserted through the anus to display the anastomotic region, including the blind limb of side-to-end anastomoses. Electronic photo documentation was acquired.

### Target condition

The target condition was defined as anastomotic leak or fistula originating from the anastomosis. The target condition should be asymptomatic at the time of testing, but potentially harmful in case of ileostomy reversal, thus clinically relevant. Asymptomatic was defined as not receiving therapy for an AL, and the absence of symptoms indicative of anastomotic failure such as fever, abdominal or (peri-) anal pain, and discharge of pus or blood.

### Reference standard

No generally accepted reference standard exists for the detection of asymptomatic leaks prior to ileostomy reversal. To calculate test accuracy measures, the clinical course after ileostomy reversal served as reference standard. Uneventful reversal was regarded as a true negative result. An anastomotic leak or fistula that required any kind of therapy after reversal was regarded as true positive. For patients who were tested positive and thus did not undergo ileostomy reversal as planned, accordance of CE and FE was regarded as true positive. In case of discordant results, patients’ history and files were reviewed for symptoms indicative of AL.

### Eligibility criteria and enrolment

All patients that had received a LAR for rectal cancer at the University Hospitals Mannheim (2013–2015) and Dresden (2005–2017) were retrospectively reviewed. No exclusions were made, and samples of both centers consisted of consecutive patients. Two groups were formed according to status of previous AL. For definition of AL, the grading system proposed by the International Study Group of Rectal Cancer (ISGRC) was used [17]. Clinically relevant AL after LAR was defined as ISGRC leak grade B (leak requiring only conservative treatment) or grade C (leak requiring operative intervention). ISGRC leak grade A (radiologic leak or leak without need for therapeutic intervention) was not regarded as a clinically relevant AL after LAR for this assessment. Patients with previous AL Grade B or C after LAR were included in group 1. Patients without clinically apparent AL after LAR were included in group 0.

Due to a low case count in group 1, the recruitment interval for group 1 was increased to 2009–2015 for the University Hospital Mannheim.

All analyzed patients had CE and FE routinely performed prior to ileostomy reversal.

### Surgery

Rectal resection was performed as LAR with total mesorectal excision (TME) and routine formation of a loop ileostomy. The colorectal anastomosis was fashioned as side-to-end anastomosis using a circular stapler, with a blind stump of 2 cm (Mannheim collective). In Dresden, an end-to-end anastomosis using a circular stapler was constructed.

### Timing of tests

Without postoperative complications, ileostomy reversal was usually planned 3 to 4 months after LAR with outpatient FE and CE prior to reversal. In case of postoperative complications, the timing of the reversal depended primarily on the healing process. Delays of several weeks to months were customary in those cases.

### Digital photo documentation

Clinic Win Data, version 8.08.0010, E&L medical systems GmbH, Erlangen, Germany, was used for digital photo documentation of endoscopic imaging. Syngo.share view diagnostic, software version VA26A, ITH icoserve technology for healthcare GmbH, Innsbruck, Austria, was used for radiologic imaging documentation.

### Statistical analysis

Statistical analysis was performed with IBM® SPSS® Statistics Version 25, SPSS Inc., Chicago, IL. Continuous variables are reported as medians with interquartile range (IQR), whereas categorical variables are reported as total numbers with group-related percentages. Chi-square test was used to analyze differences between categorical variables; for continuous variables, Student’s *t* test was used. A *p* value of < 0.05 was considered statistically significant. Diagnostic accuracy measures were calculated from the respective 2 × 2 contingency tables.

## Results

Two hundred seven consecutive patients who underwent LAR and had shown no clinical sign of postoperative AL were identified and assigned to group 0, whereas 86 patients had suffered from clinically relevant AL after initial LAR and were assigned to group 1. Median age did not differ significantly between groups 0 and 1 (62 years vs. 61 years, *p* = 0.477); however, there were significantly more male patients in group 1 than in group 0 (80% vs. 59%, *p* = 0.001) (Table 1). No patient suffered from complications of FE or CE that required medical, interventional, or surgical therapy. In one case of antegrade CE, initial incorrect irrigation of the oral limb was reported; however, no complications resulted from this.

**Table 1:** Group characteristics

	*All patients*	Group 0	Group 1	p value
Characteristics	LAR + stoma	No leak after LAR	Anastomotic leak after LAR	
*n*	293	207	86	
Female sex [%]	34	41	20	
Median age (years) (IQR^a^)	62 (54–69)	*62 (54–69)*	61 (54–68)	0.477

^a^Interquartile range

### Overall diagnostic test accuracy

Sensitivity for detection of AL of FE was 76% and specificity was 100%. Sensitivity of CE was 60% and specificity was 100% as well. The positive predictive value for both tests was 100%, and negative predictive value was 98% for FE and 96% for CE. There was a calculated negative likelihood ratio of 0.24 found for FE and 0.40 for CE. Due to no false positive test results, the positive likelihood ratio and the diagnostic odds ratio could not be calculated. Overall and group-related contingency tables and accuracy values of FE and CE are displayed in Table 2.

**Table 2:** Diagnostic accuracy measures of flexible endoscopy (FE) and contrast enema radiography (CE)

Cohort	*n*	Test	TP	FP	FN	TN	Sens (%)	Spec (%)	PPV (%)	NPV (%)	-LR	Prev (%)	NNT
All patients	293	FE	19	0	6	268	76	100	100	98	0.24	8.5	15
		CE	15	0	10	268	60	100	100	96	0.40		20
Group 0	207	FE	2*	0	1	204	67	100	100	100	0.33	1.4	104
		CE	2*	0	1	204	67	100	100	100	0.33		104
Group 1	86	FE	17	0	5	64	77	100	100	93	0.23	25.6	5
		CE	13	0	9	64	59	100	100	88	0.41		7

*FE* flexible endoscopy, *CE* contrast enema radiography, *TP/FP/FN/TN* true/false positives/negatives for detection of anastomotic leak, *Sens* sensitivity, *Spec* specificity, *PPV/NPV* positive/negative predictive value, *-LR* negative likelihood ratio, *Prev* prevalence of AL, *NNT* number needed to test to find one anastomotic leak. Due to no false positive findings, positive likelihood ratio and diagnostic odds ratio could not be calculated

*One patient with pathological findings before ileostomy closure received vacuum sponge therapy, and one patient had a fistula that healed spontaneously. After completion and negative diagnostic testing, the ileostomy was eventually reversed uneventfully in both cases

### Group 0 (no clinical leakage after LAR)

There was complete accordance of CE and FE findings in group 0.

One patient with concordant pathological findings before ileostomy reversal received vacuum sponge therapy, and one patient had a fistula that healed spontaneously. After completion and negative diagnostic testing, the ileostomy was eventually reversed uneventfully in both cases.

One patient suffered from anastomotic leak after ileostomy reversal, although both examinations had been negative.

Both tests had a sensitivity of 67% and a specificity of 100.0% in this group. The prevalence of AL prior to ileostomy reversal was 1.4%, and the number needed to test (NNT) for detection of one AL before ileostomy reversal for both tests was 104 (Table 2).

### Group 1 (clinically relevant leakage after LAR)

Accordance of CE and FS in group 1 was 95% (82 out of 86).

Of the 69 patients with concordant negative findings prior to ileostomy reversal, 5 (7%) had postoperative complications related to the anastomotic site (4 anastomotic leaks and 1 rectovaginal fistula). Neither FE nor CE had preoperatively shown pathological findings in these patients; thus, they were false negative results. Sixty-four patients in group 1 had their ileostomy reversed uneventfully after concordant negative tests, and the test results were recorded as true negatives.

Of the 13 patients with accordant pathological findings, 11 received endoscopic therapy. In the remaining 2 cases, the reversal was delayed, the anastomosis healed spontaneously, and the ileostomy was reversed after repeated tests. Thus, findings in all 13 patients were true positives.

The 4 patients with discordant findings had negative CE, but positive FE and received endoscopic vacuum sponge therapy for detected leaks. Thus, FE had shown 4 true positives and CE 4 false negative results.

In group 1, FE had superior sensitivity to CE (77% vs. 59%) with the same specificity of 100%. The prevalence of a pathological anastomosis prior to ileostomy reversal was 25.6%. The number needed to test for detection of one clinically relevant AL before ileostomy reversal in this group was 5 for FE and 7 for CE (Table 2).

## Discussion

This study assessed the diagnostic test accuracy of CE and FE for the detection of asymptomatic anastomotic leak. It could be demonstrated that routine CE has no advantage over FE in testing the anastomosis. Even in group 1, with a higher prevalence of asymptomatic AL, CE did not add clinically relevant information to FE. Radiation exposure and discomfort for patients caused by CE are further arguments against this test. Other uses for water-soluble CE such as prediction of fecal incontinence seem to be of little value [5, 18]. The abolishment of routine CE in favor of endoscopic techniques such as FE appears to be reasonable. In light of the current evidence base, holding on to CE in patients with higher risk for a leak can be arguable for safety reasons. However, the reported high negative predictive value (98.4%) of CE [2] has only been demonstrated in mixed patient cohorts. In patients who had a complicated postoperative course after LAR, diagnostic accuracy of CE has not been calculated before. This study showed that FE is more sensitive than CE in this important patient cohort. Direct imaging of the anastomosis with the option to clean the anastomotic site of fibrin coating and probing pouches are possible reasons for the superiority of FE over CE. However, FE might not be available to all surgeons. There are no studies formally addressing this problem. Thoroughly executed clinical and imaging examinations are of critical importance in this setting. When opting for CE, the evaluation by the operating surgeon with full knowledge of the postoperative anastomotic anatomy is advisable. Additional clinical investigation of the anastomosis by digital rectal examination has not been part of this investigation. It has been demonstrated that digital rectal examination can compare favorably to CE [19]. Being a quick to perform clinical test, it should always be included in the decision-making before ileostomy reversal.

We could add new knowledge by demonstrating the relevance of previous AL on test accuracy for AL in asymptomatic patients and AL prevalence. In group 0, there were only 1.4% pathologic anastomoses compared with 25.6% in group 1, and 104 patients in group 0 had to be tested to find one AL. It has previously been shown that routine testing adds no information in patients without postoperative complications [9, 20], and ileostomies could be safely reversed even in cases of a radiologic leak [4]. Future investigations are needed to weigh the high effort of routine testing against its gain of information in larger cohorts.

The asymmetrical distribution of gender in the whole study cohort might be attributed to a higher risk for colorectal cancer in men. The higher percentage of men in group 1 compared with group 0 supports a previously reported higher risk for AL in men [10]. The two groups concur in median age.

### Limitations of this study

One limitation of this study is its retrospective design. Also, to increase the sample size in group 1 (leak after rectal resection), the recruiting interval for this group was prolonged in one center; thus, time-related influences in the comparison of the groups cannot be excluded, and a precise leak rate after initial LAR cannot be presented. No standardized test reference (“gold standard”) is available for leakage in the context of ileostomy reversal. Clinical outcome after ileostomy reversal served as clinically and patient-relevant primary reference standard for true or false negative findings. Positive tests being unlikely to be reversed might thus be unrecognized as false positives, potentially overestimating the accuracy of both tests.

## Conclusion

Flexible endoscopy is the more accurate diagnostic test for the detection of asymptomatic anastomotic leaks prior to ileostomy reversal. Contrast enema showed no gain of information. In the group without complications after the initial rectal resection, 104 must be tested to find one leak prior to reversal. In those patients, routine diagnostic testing additional to digital rectal examination may be questioned.
